# Metabolic regulation of microglial phagocytosis: Implications for Alzheimer's disease therapeutics

**DOI:** 10.1186/s40035-023-00382-w

**Published:** 2023-10-31

**Authors:** Izabela Lepiarz-Raba, Ismail Gbadamosi, Roberta Florea, Rosa Chiara Paolicelli, Ali Jawaid

**Affiliations:** 1 Laboratory for Translational Research in Neuropsychiatric Disorders (TREND), BRAINCITY: Center of Excellence for Neural Plasticity and Brain Disorders, Nencki Institute of Experimental Biology, Warsaw, Poland; 2 Swiss Federal Institute of Technology (ETH), Zurich, Switzerland; 3 Department of Biomedical Sciences, University of Lausanne, Lausanne, Switzerland

**Keywords:** Microglia, Metabolism, Alzheimer's disease, Neurodegeneration, Inflammation, Phagocytosis

## Abstract

Microglia, the resident immune cells of the brain, are increasingly implicated in the regulation of brain health and disease. Microglia perform multiple functions in the central nervous system, including surveillance, phagocytosis and release of a variety of soluble factors. Importantly, a majority of their functions are closely related to changes in their metabolism. This natural inter-dependency between core microglial properties and metabolism offers a unique opportunity to modulate microglial activities via nutritional or metabolic interventions. In this review, we examine the existing scientific literature to synthesize the hypothesis that microglial phagocytosis of amyloid beta (Aβ) aggregates in Alzheimer’s disease (AD) can be selectively enhanced via metabolic interventions. We first review the basics of microglial metabolism and the effects of common metabolites, such as glucose, lipids, ketone bodies, glutamine, pyruvate and lactate, on microglial inflammatory and phagocytic properties. Next, we examine the evidence for dysregulation of microglial metabolism in AD. This is followed by a review of in vivo studies on metabolic manipulation of microglial functions to ascertain their therapeutic potential in AD. Finally, we discuss the effects of metabolic factors on microglial phagocytosis of healthy synapses, a pathological process that also contributes to the progression of AD. We conclude by enlisting the current challenges that need to be addressed before strategies to harness microglial phagocytosis to clear pathological protein deposits in AD and other neurodegenerative disorders can be widely adopted.

## Background

Microglia, the resident immune cells of the central nervous system, are implicated in several key processes integral to brain homeostasis [1–3]. Microglia continuously survey the brain parenchyma for removal of apoptotic and necrotic cells, toxic protein aggregates, and pathogens besides releasing critical growth factors [4]. Notably, inflammatory response and phagocytosis are among the most crucial properties of microglia that are not only important for their physiological functions but also relevant to their implication in brain pathologies.

Microglia are highly heterogeneous and equipped with an extensive repertoire of surface receptors, which allow them to detect changes in the brain environment [5]. Upon exposure to cytokines and other environmental stimuli, microglia readily acquire different phenotypes that are characterized by varying levels of inflammatory response [6]. Additionally, phagocytosis, a function microglia share with other tissue macrophages, is critical to their role in brain disorders. Phagocytosis is initiated by the interaction of target particles with phagocytic receptors expressed on microglia. Upon recognition of targets, microglia undergo extensive cytoskeletal rearrangement that allows the engulfment of particles. This is followed by the assembly of the phagolysosome, which involves the fusion of the phagosome containing the phagocytosed particle with the lysosome. The engulfed particles are eventually digested by the lysosomal enzymes [7, 8].

**Fig. 1:**
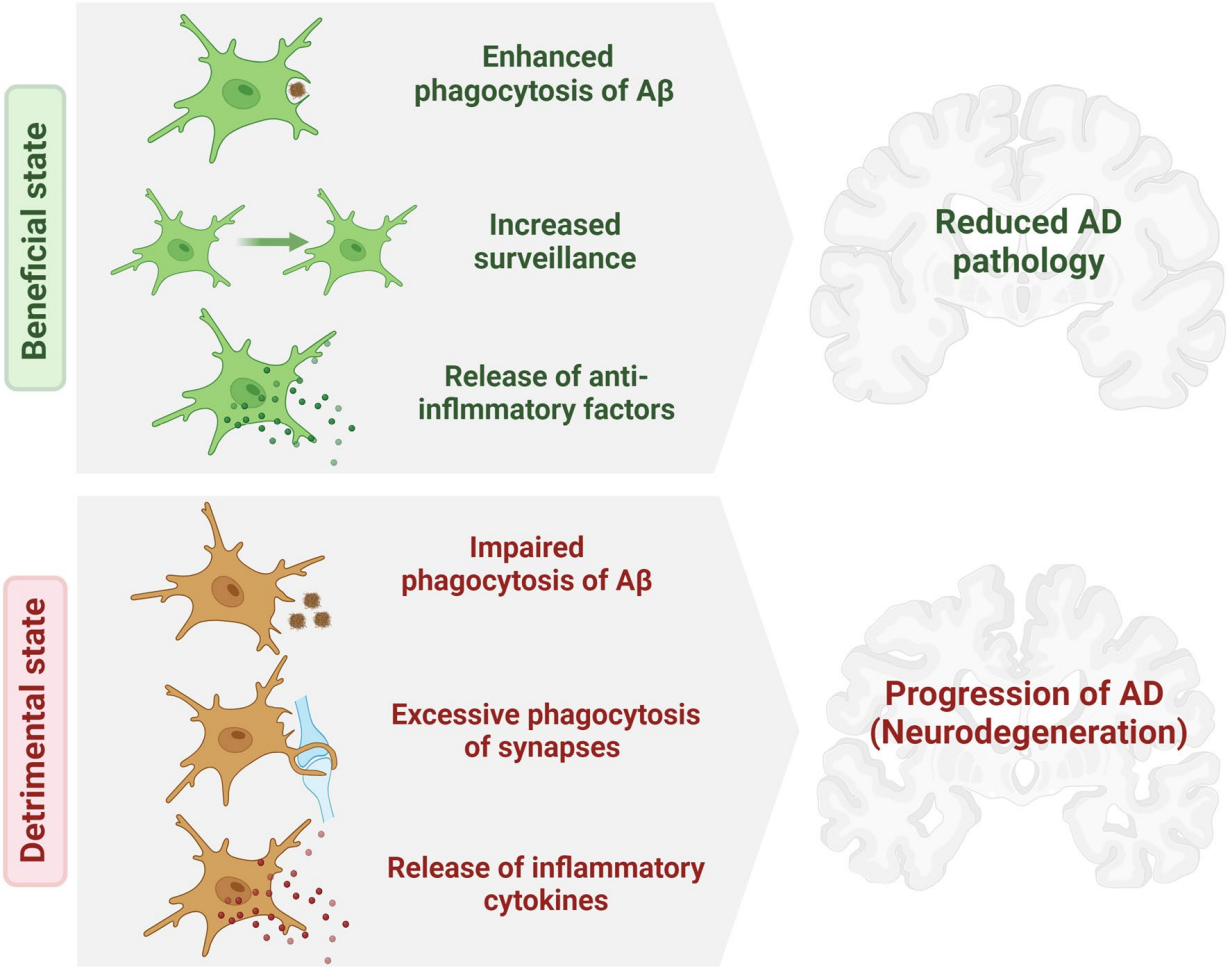
Two faces of microglial functions in AD. Microglia can be protective in AD when they efficiently recognise, engulf and degrade Aβ, maintain their homestatic surveillance functions and secrete anti-inflammatory factors. However, microglia can also contribute to progression of AD pathology and neurodegeneration when they exhibit impaired phagocytosis of Aβ, excessively phagocytize synapses and processes of live neurons and release inflammatory cytokines

Owing to their phagocytic and inflammatory functions, microglia are increasingly being recognized as key players in the pathogenesis and pathophysiology of neurodegenerative disorders (NDDs), such as Alzheimer’s disease (AD). AD, which is the most common cause of dementia, is characterised by a series of pathological hallmarks: the early extracellular aggregation of amyloid beta (Aβ), followed by neuronal aggregation of hyperphosphorylated tau protein [9]. These two events are simultaneously accompanied by progressive synaptic damage and neurodegeneration that eventually culminate into substantial brain atrophy. Microglia can impact the progressive pathology of AD bidirectionally [10, 11]. By clearing toxic Aβ aggregates, microglia can counter the progression of AD and help in the restoration of brain homeostasis. However, uncontrolled and non-selective phagocytosis of healthy synapses by microglia could contribute to neurodegeneration [12]. Additionally, an aberrant response of microglia to Aβ deposits and degenerating neurons can lead to inflammatory states that further contribute to neuronal damage [3] Thus, finding ways to preferentially enhance microglial phagocytosis of toxic deposits such as Aβ without degradation of healthy synapses or exaggerated inflammatory responses could be an effective preventive and therapeutic strategy in NDDs, such as AD (Fig. 1).

Emerging evidence now suggests that microglial functions are closely tied to the adaptation of their metabolism in response to environmental stimuli. Such metabolic programming leads to altered regulation of microglial inflammatory and phagocytic responses, hence impacting their role in health and disease [13–15]. More recent evidence further suggests that manipulation of metabolic regulators in microglia can alter microglial phagocytosis. Notably, genetic deletion or pharmacological inhibition of hexokinase 2 (HK2) protein that regulates the first rate-limiting step in glycolysis increases Aβ clearance by microglia via upregulating lipid metabolism in AD mice [16]. Moreover, the depletion of TAR DNA-binding protein of 43 kDa, a protein that regulates cellular and whole-body metabolism, was shown to increase Aβ clearance by microglia accompanied with enhanced synaptic pruning both in vitro and in vivo [17]. Furthermore, starvation or inhibition of the insulin/insulin-like growth factor 1 nutrient signalling pathway in the microglia similarly increases phagocytosis of Aβ [18]. These studies provide intriguing evidence that microglial phagocytic activity can be controlled by targeting their metabolism and highlight the potential of metabolic manipulations to refine the protective role of microglia in AD and potentially other NDDs.

This review aims to comprehensively examine how different nutrients such as glucose, lipids, ketone bodies, pyruvate, lactate and glutamine alter microglial inflammatory and phagocytic functions. Furthermore, emphasis is placed on metabolic dysregulation of microglia in AD and the potential of metabolic interventions in altering microglial functions in vivo. Finally, a role for metabolic factors in the regulation of pathological phagocytosis of healthy synapses by microglia is evaluated to elucidate strategies that can enhance microglial clearance of Aβ in AD while minimizing their inflammatory outputs and excessive removal of healthy synapses.

## Metabolic flexibility of microglia

Microglia are highly plastic immune cells with the ability to alter their functions and phenotypes in response to environmental stimuli. This adaptability is closely aligned with their flexibility to utilize various energy substrates, including glucose, amino acids, lipids, ketone bodies, lactate and pyruvate [19–21]. Notably, these cells are distinguished by the vast range of nutrients they can metabolize to meet their dynamic functional demands and adaptation between glycolysis and oxidative phosphorylation (OXPHOS) [22–27].

Microglia are known to rapidly shift their metabolism between OXPHOS and glycolysis under specific conditions [21, 28–30]. Under homeostatic conditions, microglia mostly rely on OXPHOS for their energy demand [28, 29]. OXPHOS synthetises adenosine triphosphate (ATP) by phosphorylation of adenosine diphosphate (ADP) through the electron transport, which takes place in the mitochondria during aerobic respiration. This process creates reactive oxygen species (ROS) as a natural by-product, which in normal conditions are balanced by antioxidant systems, such as glutathione. However, microglia can shift to a preferential reliance on glycolysis for ATP production under several pathophysiological conditions, such as neurodegeneration. Glycolysis enables a faster, albeit less efficient rate of ATP production. Moreover, glycolytic intermediates can also divert into the pentose phosphate pathway to generate precursors for nucleotide and amino acid biosynthesis, several of which are critical for production of cytokines [21, 28].

Metabolic flexibility is emerging as a key characteristic of microglia. It allows their functional viability and adaptability in case of nutrient depletion or alteration, which is crucial to their role in maintaining homeostasis in the brain. The brain is the most energy-demanding organ in the body, which is vulnerable to fluctuation of energy substrates due to a variety of conditions, such as prolonged fasting, ischemia, or diabetes mellitus (DM) [31]. The metabolic flexibility of microglia facilitates their normal range of functions under such fluctuating conditions [30]. However, the dependence of microglial functions on metabolic signals also renders them sensitive to metabolic disorders and nutritional insults [32]. In the next section, we evaluate the existing literature about how different energy substrates shape microglial functions, notably their inflammatory outputs and phagocytosis.

## Dynamics of microglial metabolism and its interaction with microglial inflammatory outputs and phagocytosis

Owing to their aforementioned metabolic flexibility, microglia are able to utilize different metabolic substrates based on the accessibility of nutrients as well as their specific functional demands. This section focuses on how microglia utilize glucose, lipids, ketone bodies, glutamine as well as metabolites of glucose such as pyruvate and lactate (Table 1). It is critical to note that a variety of in vitro microglia models (Table 2) have been used in the studies discussed below, which could explain some of the discrepancies observed.

**Table 1:** Effects of individual nutrients on microglial phagocytosis

Nutrient	Manipulation	Effect on phagocytosis	Phagocytosed particle	Proposed mechanism	Microglial model	References
Glucose	Glucose starvation (use of glucose-free medium)	↑	Fluorescent beads	Decrease in oxidative phosphorylation	Primary rat microglia	[40]
	Oxygen–glucose deprivation	↑	Fluorescent latex beads and myelin debris	Activation of RhoA/ROCK signalling; increased expression of complement receptor 3, CD11b, SR-A and ATP	Primary rat microglia	[43]
	Treatment with deoxyglucose	↑	Carboxylate-modified latex microspheres	Depletion of ATP production	Primary rat microglia and BV2 microglia	[41]
	Treatment with an inhibitor specific for GLUT1 (STF31)	↓	pHrodo Green* S. aureus* BioParticles	Decrease LPS + IFNγ-induced expression of TNFα, IL-1β, IL-6, and CCL2, iNOS; reduced IL-4-induced expression of Arg1	B6M7 microglia and primary mouse microglia	[35]
	Genetic ablation of hexokinase 2	↑	Carboxyfluorescein-labelled Aβ42	Upregulation of LPL expression, increase in lipid metabolism	Primary mouse microglia	[16]
			Microparticles (sulfate microspheres)	Impaired mitochondrial function	Primary mouse microglia	[42]
Lipids	α-linolenic acid supplementation	↑	Tau monomers and aggregates	Repolarization of axis of microtubule organizing center to facilitate microglial migration	N9 microglia	[55, 56]
	Docosahexaenoic acid (DHA) and eicosapentaenoic acid (EPA) supplementation	↑	Cy-3-labelled myelin	Reduced expression of TNFα and nitric oxide; increased expression of CD206 and/or TGF-β	Primary mouse microglia	[54]
			Aβ42	Decreased expression of CD40 and CD86	CHME3 microglia	[57]
Ketone bodies	β-hydroxybutyrate treatment	↑	Nile Red FluoSphere with 1-mm diameter microspheres	Activation of Akt-small RhoGTPase	BV2 microglia	[14]
Pyruvate	Ethyl pyruvate treatment	↑	CFSE-conjugated myelin debris	Upregulation of Sox2	BV2 and primary mouse microglia	[79]
Lactate	Lactate supplementation	↑	FITIC-Dextran	Increase of CD68 expression	Primary rat microglia	[78]
		↓	Fluorescent latex beads and pHrodo Red-labelled Aβ42	HCAR1 activation	HMC3, N9 and primary mouse microglia	[80]

*HCAR1* Hydroxycarboxylic acid receptor 1; *iNOS* Inducible nitric oxide synthase

**Table 2:** Brief description of the commonly used in vitro microglial models

Species	Microglia cell lines	Description	References
Mouse	BV2	Derived by immortalizing neonatal C57/BL6 murine microglia	[86, 117, 166, 174]
	B6M7	Immortalized from primary microglia isolated from C57BL/6 J mice brain	[35]
	N9	Derived by immortalizing embryonic mouse brain microglia by v-mil oncogenes	[175, 176]
	SIM-A9	Spontaneously Immortalized Microglia-A9 cell line (SIM-A9) from a primary glial culture of postnatal murine cerebral cortices	[177]
	Primary cells	Derived from mouse pup postnatal brain tissues, plated and cultured to obtain adherent microglia culture	[35, 54]
Human	HMC3 (CHME3, CHME5)	Derived by transfecting primary human embryonic microglial cells with the SV40 large T antigen	[57, 65, 178]
	iPSCs-derived microglia	Obtained via reprogramming human fibroblasts (or other cells) into induced pluripotent stem cells (hiPSC) followed by differentiation into microglia by providing key microglial growth factors and signaling molecules	[170, 179]
	Primary	Isolated from brain specimens obtained at autopsy or at surgery. Commercially available primary human microglia are obtained from CNS-Cortex	[180]
Rat	Primary	Obtained from postnatal brain tissues of rats, plated and cultured to obtain adherent microglia culture	[181]

### Glucose

**Fig. 2:**
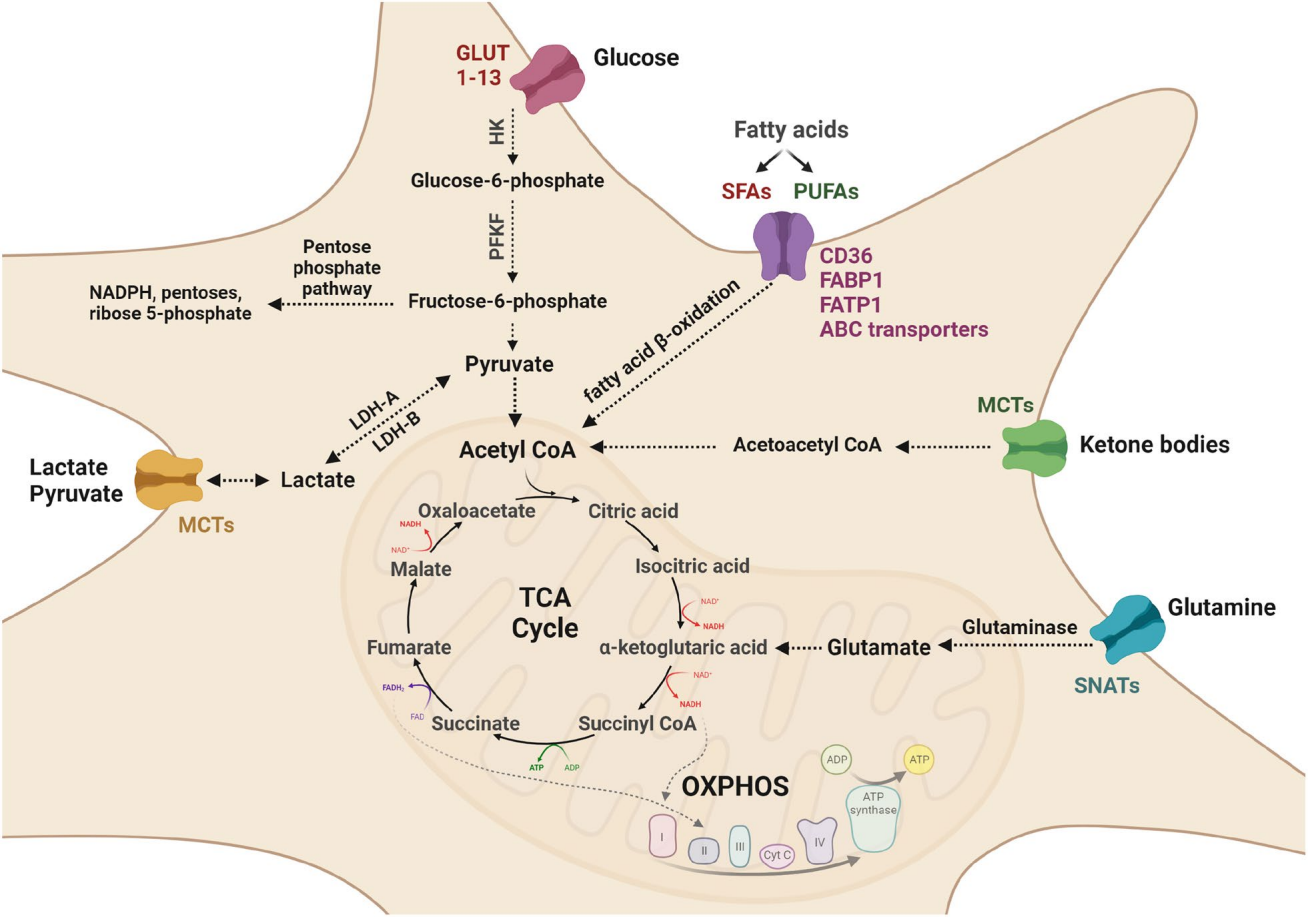
Dynamics of microglial metabolism. Microglia have the ability to utilize several nutrients for energy production. Uptake of glucose in micorglia is facilitated via glucose transporters (GLUTs), which then undergoes glycolysis to produce pyruvate. Pyruvate is converted into acetyl co-enzyme A (acetyl-CoA) that is shunted into the tricarboxlic acid cycle (TCA). TCA cycle produces nicotinamide adenine dinucleotide (NADH) for oxidate phosphorylation (OXPHOS) generating ATP. Parallel to glycolysis and OXPHOS, glucose taken up by the microglia can also produce NADPH and ribose 5-phosphate through the pentose phosphate pathway. Several other nutrients, such as fatty acids, lactate, pyruvate, and glutamine supplement ATP production in microglia through direct entry into the TCA cycle and thus can enhance OXPHOS even in the absence of a constant supply of glucose

The brain is a unique energy-demanding organ in the human body–it accounts for only 2% of total body weight, yet consumes about 20% of the overall energy [33]. Under normal circumstances, glucose is the major energy source for the brain and its resident cells including microglia [34]. Subsequent to uptake or production via gluconeogenesis, glucose undergoes glycolysis to generate pyruvate and yields 2 ATP molecules. Under aerobic conditions, pyruvate enters the tricarboxylic acid (TCA) cycle, leading to the production of nicotinamide adenine dinucleotide and flavin adenine dinucleotide FADH2. These reducing equivalents contribute to mitochondrial OXPHOS, generating further 36 ATP molecules [34] (Fig. 2).

Microglia express various glucose transporters (GLUTs), with GLUT1 being predominantly responsible for glucose uptake and integral for several microglial functions [40]. Pharmacological inhibition of GLUT1 in BV2 microglia reduced glucose uptake, suppressed extracellular acidification rate that indicates impaired glycolytic efficiency, and dampened microglial inflammatory response to lipopolysaccharides (LPS) and interferon gamma (IFNγ) [35]. Also, inhibition of GLUT5 in murine SIM-A9 microglia-like cells, as well as in primary mouse microglia, attenuates pro-inflammatory gene expression [36]. Furthermore, fluctuations in glucose concentration were found to affect the inflammatory state of BV2 microglia in culture; low-to-high glucose shift increased expression of tumour necrosis factor alpha (TNF-α), inducible nitric oxide synthase, and cyclooxygenase 2, whereas high-to-low shift promoted autophagy and apoptosis [37]. These findings underscore the significant role of glucose metabolism in regulating microglial inflammatory functions.

Various studies have further demonstrated that inflammatory triggers such as LPS, LPS/IFNγ, Aβ and interleukin-1 beta (IL-1β)/IFNγ lead to metabolic reprogramming of BV2 and primary mouse microglia with a shift from OXPHOS to glycolysis as the preferred pathway for ATP production [21, 38, 39]. As glycolysis also enables the production of intermediates for inflammatory mediators [29], changes in microglial glycolysis have been shown to regulate microglial inflammatory response. Notably, a recent study showed that inhibition of glycolysis suppressed BV2 microglial transcriptomic response to LPS by preventing the activation of transcription factor nuclear factor kappa B (NF-κB) [38].

With a few exceptions, reducing glucose metabolism has also been shown to enhance microglial phagocytosis. Glucose deprivation in primary rat microglia is associated with increased phagocytosis of latex beads [40]. Furthermore, reducing glucose metabolism via pharmacological inhibition of glycolysis in primary rat microglia was shown to enhance phagocytosis of microspheres [41]. Moreover, microglia-specific genetic ablation of glycolytic enzyme HK2 leads to enhanced phagocytosis of Aβ42 [16] and microspheres [42]. Similarly, oxygen–glucose deprivation enhances phagocytosis of latex beads and myelin debris in primary rat microglia [43].

Taken together, the ability of microglia to adapt their metabolic pathways based on fluctuations in glucose levels has significance in regulating their responses to different physiological and pathological contexts. Glucose metabolism thus is emerging as a dynamic orchestrator of microglial behaviour, offering potential avenues for therapeutic intervention in AD and other NDDs.

### Lipids

Lipids constitute another important energy substrate for microglia. Lipid molecules consist of repeating units of fatty acids (FAs); long-chain hydrocarbons that can be distinguished into two main categories: saturated and unsaturated fatty acids. Saturated fatty acids (SFAs) are straight-chain organic acids, while unsaturated fatty acids contain at least one double bond between the carbon atoms. Unsaturated fatty acids can be further categorized into monounsaturated fatty acids, which have one double bond and polyunsaturated fatty acids (PUFAs), which have two to six double bonds. Examples of PUFAs include arachidonic acid (AA), docosahexaenoic acid (DHA), eicosapentaenoic acid (EPA) [44].

Fatty acid β-oxidation (FAO) provides up to 20% of the total brain energy requirement [45]. FAO is preceded by either passive diffusion of FAs into the mitochondria or their transport via carnitine shuttle or peroxisome oxidation depending on their chain length. Long-chain FAs undergo carnitine shuttle from the cytoplasm where FAs are metabolised by fatty acyl-CoA synthetase to fatty acyl-CoA, allowing its diffusion into intermembrane mitochondrial space. The enzyme carnitine acetyltransferase 1 (CPT1) then converts fatty acyl-CoA to fatty acylcarnitine. Subsequently, fatty acylcarnitine is transported across the inner mitochondrial membrane and converted back to fatty acyl-CoA, which is oxidized by the β-oxidation enzymatic machinery of the mitochondrial matrix to produce acetyl-CoA. Acetyl-CoA is then supplied to the TCA cycle [46]. Oxidation of very-long-chain FAs, on the other hand, is conducted in the peroxisome after conversion of free FAs into CoA esters, which are transported to the peroxisome via ATP-binding cassette transporters subclass D (ABCD) transporters. Then, peroxisomal acyl-coenzyme A oxidase catalyses β-oxidation, which consequently also yields acetyl-CoA, eventually feeding the TCA cycle [47] (Fig. 2).

Microglia express various fatty acid transporter proteins, including class B scavenger receptors (SR) such as CD36, ABCD transporters, fatty acid-binding proteins (FABPs) and fatty acid transportation proteins [47–49] that allow uptake of fatty acids. However, the transport of fatty acids from the cytoplasm to mitochondria in microglia seems to have species-specific differences. Notably, it has been suggested that CPT1a is present in mouse but not in rat microglia [21, 46].

Similar to glucose, FAs can also regulate the inflammatory properties of microglia. SFAs have been shown to act as ligands for toll-like receptors (TLRs), which are usually reserved for pathogen recognition and thereby promote pro-inflammatory gene expression in BV2 and primary mouse microglia via activation of transcription factor NF-κB [50]. On the other hand, extensive literature indicates that PUFAs exert an anti-inflammatory effect in microglia both in vitro and in vivo [51–54]. Moreover, primary mouse microglia stimulated with IL-1β or IFNγ exhibit reduced expression of genes coding for enzymes involved in mitochondrial and peroxisomal β-oxidation. Conversely, microglia stimulated with the cytokine IL-4, known to elicit anti-inflammatory properties, show enhanced mitochondrial β-oxidation [21].

The evidence for regulation of microglial phagocytosis by lipids is also rapidly accumulating with several studies on the effects of PUFAs and SFAs. Incubation with α-linolenic acid (ALA) increased phagocytosis of extracellular tau monomers and aggregates in a study on mouse N9 microglia [55, 56]. In addition, omega-3 acids derived from ALA, such as EPA and DHA, could also impact microglial phagocytosis. Human immortalized microglia and primary mouse microglia incubated with DHA or EPA showed a decrease in pro-inflammatory markers and an increased phagocytic activity against Aβ [54, 57].

In conclusion, studies on cultured microglia suggest a potential dually beneficial role of PUFAs in AD with a decrease in microglial inflammatory output and enhancement of Aβ phagocytosis.

### Ketone bodies

In order to support their bioenergetic homeostasis, microglia can also metabolize ketone bodies such as acetoacetate and β-hydroxybutyrate (βHB). Under non-fasting conditions, blood levels of ketone bodies are usually low, which contribute to less than 5% of the brain energy demands. However, prolonged fasting increases the level of ketones considerably, which then replace glucose as the main energy source of the brain contributing to almost 60% of the brain energy requirements [58–60]. Ketone bodies are transported across the cell membrane via monocarboxylate transporters (MCTs) [61] and subsequently metabolised to release two molecules of acetyl-CoA, which can generate energy by entering the TCA cycle [60] (Fig. 2).

Ketone bodies have been shown to suppress microglial pro-inflammatory phenotype by decreasing the production of inflammatory cytokines TNFα, IL-1β, IL-6, nitrite and ROS [62–64]. Moreover, in vivo and in vitro studies demonstrated that βHB can induce microglial ramification and expression of anti-inflammatory genes, including IL-10 and cluster of differentiation 206 (CD206) [14]. However, in another study, βHB was shown to enhance the glycolytic flux in LPS-induced BV2 microglia along with increased expression of the pro-inflammatory gene *NOS2*. At the same time, βHB increased the accumulation of key immunometabolites, such as α-ketoglutarate and fumarate generated by the TCA cycle [65], which are known to possess anti-inflammatory properties [66–69]. Therefore, it is plausible that βHB has a potential immunomodulatory effect on microglia, which can initially promote their inflammatory response but then moderate it through enhanced production of anti-inflammatory immunometabolites. Some preliminary evidence supports that ketone bodies may also be involved in the regulation of microglial phagocytosis. Stimulation of primary mouse microglia with βHB led to microglial ramification and polarization towards an anti-inflammatory phenotype along with increased phagocytosis of beads [14].

### Lactate/pyruvate

Lactate and pyruvate, which are produced as intermediates of glucose metabolism, may also be used as energy substrates by microglia. The normal brain lactate concentration in extracellular space is 2–5 mM, which can support up to 10% of brain energy metabolism [70, 71]. Lactate is produced from pyruvate, the end product of glycolysis, under anaerobic conditions [72]. In addition to intracellular production, lactate and pyruvate can also be taken up by microglia via MCTs. While pyruvate can directly serve as a substrate for gluconeogenesis or for the TCA cycle, lactate first requires reduction to pyruvate via the redox enzyme lactate dehydrogenase B (LDHB) [72]. Notably, *LDHB* is among the most abundant genes expressed in microglia, and its expression level is considerably higher compared to those in other brain cells [73].

Lactate production was found to be increased in LPS-exposed BV2 microglia and decreased in IL-4-stimulated microglia [74, 75]. Hence, it seems that microglia in pro-inflammatory state produce more lactate because of the metabolic shift from OXPHOS to glycolysis. This increase, however, may eventually lead to a moderation of the microglial inflammatory response. Supplementation with exogenous lactate has been shown to suppress LPS-induced expression of pro-inflammatory cytokines in BV2 cells [76]. Similarly, intracerebroventricular injection of lactate ameliorated LPS-induced inflammatory profile of microglia in mice [77]. Therefore, lactate administration could also be considered as a potential strategy for modulating microglial cytokine production.

Similar to ketones, the evidence about the regulation of microglial phagocytosis by pyruvate and lactate is scarce. Stimulation of primary rat microglia with lactate was shown to enhance phagocytosis of fluorescein isothiocyanate–dextran [78]. Similarly, stimulation of primary mouse and BV2 microglia with ethyl pyruvate promoted an anti-inflammatory phenotype with increased phagocytosis of myelin debris [79]. On the contrary, another study that employed three different microglial models (primary mouse, N9, and HMC3 microglia) showed consistent reductions of microglial Aβ phagocytosis upon lactate treatment [80]. Discrepancies in the effect of lactate on microglial phagocytosis in different models could be due to the inconsistent lactate doses, the use of different microglial models, and variability in the assays and cargoes used for assessing phagocytosis.

### Glutamine

In the absence of glucose or ketone bodies, microglia can also adapt to alternative metabolic sources such as glutamine [20], which is the most abundant free amino acid in the human body [81]. The uptake of glutamine in microglia is conducted via SNAT1 (solute carrier family 38a member 1 protein) [82]. After uptake, glutamine is deaminated by glutaminase to glutamate, which is subsequently converted to α-ketoglutarate by glutamate dehydrogenase, and then fuelled into the TCA cycle [83] (Fig. 2).

Recent evidence suggests that glutamine can be a potent alternative metabolic fuel for microglia under hypoglycaemic conditions. Glutaminolysis was shown to support the maintenance of microglial motility and damage-sensing functions during insulin-induced hypoglycemia in vivo, as well as aglycemia in acute brain slices [20]. However, glutamine availability and excess during microglial pro-inflammatory responses may have harmful effects. Upon stimulation with TNF-α, microglia readily convert glutamine to glutamate via a disproportionate increase in the acitivity of glutaminase. Importantly, this glutamate is not subsequently converted to α-ketoglutarate but is secreted, thus contributing to excitotoxicity [84]. Therefore, the conversion of glutamine to glutamate is a critical step, which depending on the microglial metabolic and inflammatory state, may be beneficial or detrimental. In the case of restricted nutrient availability, glutamine may feed the TCA cycle to support energy homeostasis. However, in inflammatory conditions, glutamine can be metabolised to glutamate by microglia, and be secreted causing excitotoxicity [85]. Interestingly, glutaminase activity has also been associated with microglial phagocytosis. Hippocampal microglia in apolipoprotein E ε4 (*APOE4*) knock-in mice show increased glutaminase activity accompanied by inefficient clearance of Aβ that could be rescued by chronic administration of JHU-083, a glutaminase antagonist [86].

**Fig. 3:**
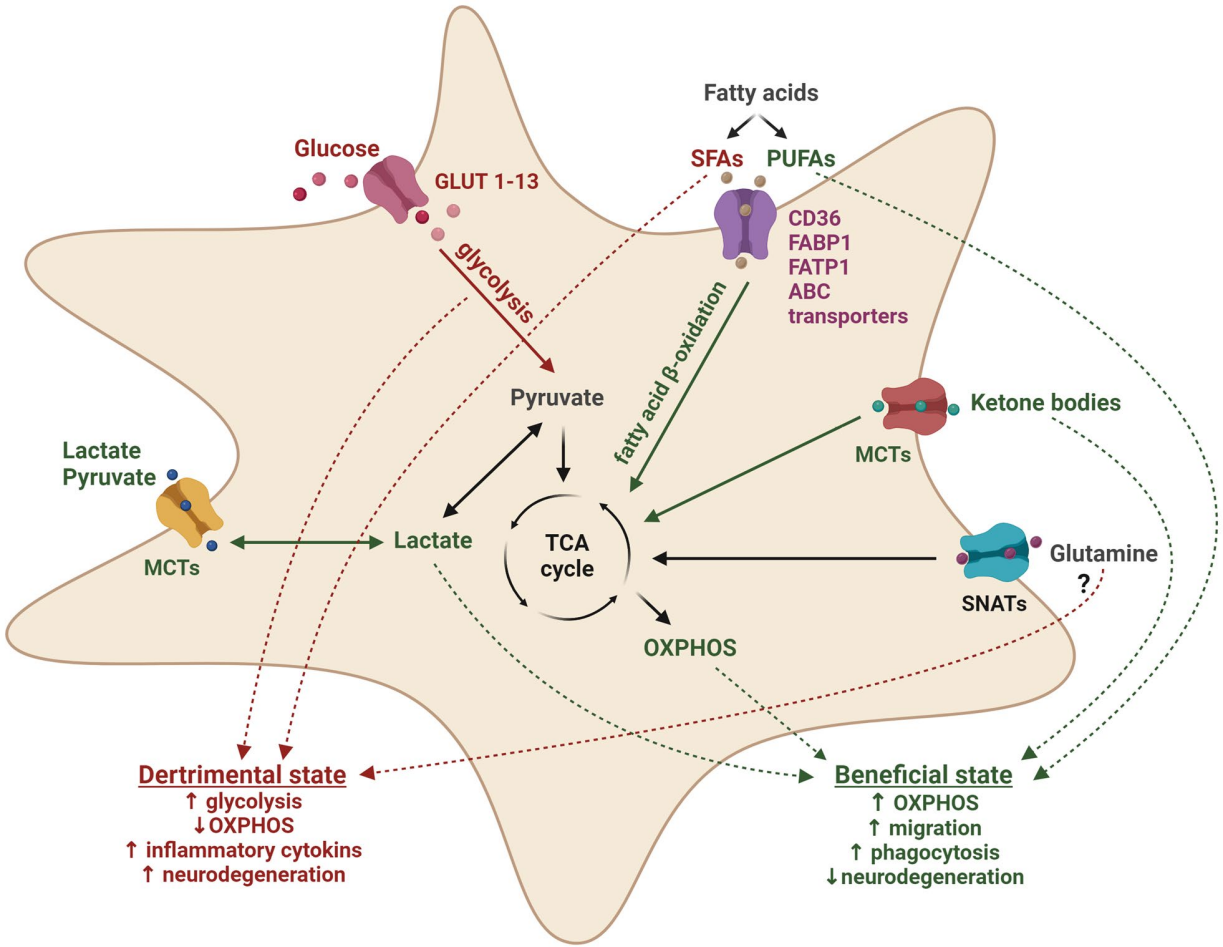
Effects of specific nutrients on microglial functions in the context of neurodegeneration. A detrimental microglial phenotype is characterized by increased production of inflammatory cytokines, enhanced glucose uptake, increased glycolysis, and reduced OXPHOS. Similar effects are likely induced by SFAs. In contrast, a beneficial phenotype of microglia is characterized by upregulation of microglial phagocytosis and repair mechanisms without a substantial increase in inflammatory cytokines. Those functions are associated with increased OXPHOS, as well as supplementation with PUFAs, ketone bodies, lactate and pyruvate

In conclusion, microglia appear to be highly metabolically flexible. They can utilize a variety of nutrients as energy substrates including glucose, lactate, pyruvate, lipids, glutamine and ketone bodies. Each of these nutrients can specifically impact microglial inflammatory response, as well as phagocytosis. The evidence supporting an enhancement of microglial phagocytosis is the strongest for PUFAs with preliminary evidence supporting a similar role for pyruvate, lactate, and ketone bodies. On the contrary, glucose, as well as specific processes involved in glucose metabolism, such as glycolysis, are associated with increased microglial reactivity and inefficient phagocytosis (Fig. 3).

However, a vast majority of the studies presented in this section focus on cultured microglia or microglial cell lines (Table 2), which have limited translational relevance. Therefore, it is paramount to examine specific alterations of microglial metabolism in vivo. In the next section, we evaluate the evidence for atypicalities in microglial metabolism in AD rodent models, as well as their comparative analysis with human patients in a few cases.

## Alterations of microglial metabolism in AD

Emerging evidence has now revealed that dysregulated microglial metabolism is a characteristic of AD. Microglial metabolism in AD is characterized by a glycolytic shift in energy production, impaired mitochondrial OXPHOS, as well as abnormalities in lipids.

Some early suggestions for microglial metabolic alterations in AD were based on genome-wide association studies that identified an increased risk for AD in individuals carrying mutations or polymorphisms in genes involved in microglial metabolism, such as *TREM2*, progranulin, and *APOE*. The genetic overlap between factors regulating metabolic signalling, microglial immune functions, and AD raises the intriguing possibility that cascades controlling microglial metabolism may modify AD risk and disease progression [87, 88].

Several notable studies on amyloid precursor protein (APP) and presenilin 1 (PS1)-based mouse models of AD reveal dysregulation of microglial glucose metabolism with a detectable shift towards glycolysis. Increased glycolysis was reported in two studies involving the double transgenic APP/PS1 AD mice along with an increase in the expression of glycolytic enzymes [89, 90]. Similarly, microglia from 6-month-old 3×Tg-AD mice that additionally carry a mutation in the gene encoding tau protein showed considerably enhanced glycolysis. However, this effect was age-dependent as the glycolysis in 18-month-old 3×Tg-AD mice was comparable to controls [91]. Furthermore, microglia from 5×FAD mice that harbour 3 mutations in APP besides one each in presenilin 1 and tau, showed higher levels of free NAD(P)H, indicating a shift towards glycolysis [92]. An independent study that employed fluorodeoxyglucose positron emission tomography (FDG-PET) in 5×FAD mice in a cell-specific manner confirmed an increased glucose uptake by hippocampal microglia. This was accompanied by upregulated expression of genes coding for glucose transporters and glycolytic proteins in RNA-seq [93]. Finally, there is some evidence to suggest that the glycolytic shift in AD could be a self-perpetuating phenomenon, whereby the increased lactate production as a result of enhanced glycolysis may promote the transcription of glycolytic genes. Increased glycolytic activity in microglia surrounding Aβ-plaques in 5×FAD mice was shown to be mediated by histone lactylation, a newly described lactate-dependent histone modification, which enhances the transcription of glycolytic genes [94]. Microglia-specific ablation of pyruvate kinase PKM2 that reduces lactate production restored microglial dysfunction and improved cognitive function in this model [94].

Critically, the shift in microglial glucose metabolism has important translational implications. Notably, expression of glycolytic enzyme HK2 was found to be elevated in microglia from 5×FAD mice and AD patients [16]. Similarly, comparative FDG-PET studies in APP/PS2 mouse models and AD patients revealed microglial activation to be the main driver of region-specific changes in FDG-PET signals [95]. Furthermore, glucose uptake was found to be preferentially enhanced in microglia in comparison to astrocytes and neurons in these studies [95, 96]. Another indirect evidence for enhanced microglial glycolysis was the elevated pan lysine lactylation and histone lactylation in post-mortem brains of AD patients compared to age-matched controls without an obvious brain pathology [94]. Finally, it has been demonstrated that microglia in AD patients may be susceptible to peripheral metabolic changes. CHME5 human microglia treated with plasma from AD patients showed a reduction in mitochondrial respiration and enhancement of glycolysis, indicating their potential to act as central mediators of metabolic changes in the periphery [97].

The shift towards glycolysis in AD is accompanied by significant compromise in mitochondrial respiration. Microglia from 6-month-old 3×Tg-AD mice showed considerable leaking of protons upon metabolic flux analysis that indicates a defective mitochondrial electron transport chain. Subsequently, this led to a reduced basal and maximal respiration capacity in 18-month-old transgenic mice. [91]. There is some evidence to speculate that microglial mitochondrial toxicity in AD models could be due to the activation of microglial ATP receptors [98]. Critically, microglial mitochondrial damage and impaired OXPHOS in APP/PS1 mice are associated with defective Aβ phagocytosis [99]. Moreover, translocator protein (TSPO), a protein present on microglial outer mitochondrial membrane, is crucial for clustering of phagocytic microglia around Aβ plaques [100]. Pharmacological and genetic loss-of-function experiments further reveal an essential role for TSPO in maintaining energy supply for microglial phagocytosis [100, 101].

Further to changes in glucose metabolism, microglia in AD transgenic models also exhibit distinctive changes in lipid metabolism [102, 103]. Time-resolved proteomic characterization of microglia in APP/PS1 and APP-KI mouse models of AD revealed dynamic alterations in the expression of fatty acid transporters FABP3 and FABP5 that correlated with advanced Aβ deposition and decline in microglial functions [104]. Furthermore, targeted liquid chromatography and mass spectrometry analysis in FACS-isolated microglia identified 4 significantly decreased and 16 significantly increased analytes in APP-KI mice [96]. Finally, lipoprotein lipase (LPL) that plays a major role in lipoprotein metabolism has been implicated in the regulation of microglial phagocytosis, in both non-AD [105] and AD contexts [16, 107]. LPL expression was found to be reduced in primary as well as BV2 microglia upon exposure to Aβ. Notably, upregulation of LPL in APP/PS1 mice by CDK5 activation induced via overexpression of its co-activator p25 promoted microglial phagocytosis of fibrillar Aβ [106]. This is further corroborated by a study on 5×FAD mice where an increase in microglial LPL after microglia-specific HK2 depletion enhanced phagocytosis of Aβ [16].

Impairments in several microglial metabolic domains in AD also implicate a role for metabolic master regulators. Recent evidence points towards TREM2 as a key regulator of microglial fitness under conditions like AD where microglial phagocytosis is required on a long-term basis and is crucial to disease progression. TREM2-deficient 5×FAD mice develop defects in microglial metabolism and phagocytosis [107]. Furthermore, when faced with the challenge of removing myelin debris on a persistent basis, TREM2-deficient microglia fail to break down the cholesterol contained in myelin debris and start accumulating cholesterol esters. Critically, this microglial phenotype is similar to that observed in microglia isolated from ApoE KO mice [108].

Overall, evidence for defects in microglial metabolism in AD is rapidly accumulating with clear indications of a glycolytic shift, mitochondrial impairment, and lipid dyshomeostasis. Crucially, these defects translate into an exaggerated inflammatory response and reduced Aβ phagocytosis by the microglia. Targeting microglial metabolism could thus be a vital strategy to restore microglial homeostatic functions in AD and other NDDs. In the next section, we examine the evidence regarding the success of different metabolic manipulation strategies in restoring and/or enhancing microglial phagocytosis.

## Enhancing microglial phagocytosis in vivo via metabolic manipulation

Accumulating evidence suggests that in vivo manipulation of microglial metabolism has a direct impact on microglial phagocytosis. Enhancing mitochondrial OXPHOS via supplementation of flavonoid sodium rutin enhanced microglial phagocytosis of Aβ and ameliorated deficits in synaptic plasticity and spatial memory in APP/PS1 and 5×FAD mice [109]. Restoration of microglial OXPHOS and subsequent microglial phagocytosis also underlies the protective effects of anti-TLR2 antibodies in primary mouse microglia that were previously found to reduce Aβ plaques in APP/PS1 mice [110, 111]. Moreover, supplementation with NAD^+^ precursor, which catalyzes oxidative metabolism [112], was found to augment microglial Aβ phagocytosis, reduce neuroinflammation and alleviate cognitive deterioration in APP/PS1 mice [113].

Treatment with broad-scale metabolic regulators, such as insulin, has also been attempted to restore microglial homeostatic functions in AD models. Intranasal insulin administration in 3×Tg-AD mice decreased microglial inflammatory response and prevented synaptic loss accompanied with a reduction of Aβ load [114]. Similarly, a single intravenous injection of insulin partially rescued the accentuating effect of high-fat diet on Aβ load and cognitive functioning in 3×Tg-AD mice [115]. Similar results were achieved with intranasal application of insulin for 6 weeks in another sudy involving APP/PS1 mice [116]. These protective effects of insulin are likely a result of enhanced microglial clearance of Aβ as opposed to reduced production. Treating BV2 microglia with insulin enhanced their ability to phagocytize Aβ upon LPS stimulation [117]. Interestingly, several human trials have revealed beneficial cognitive effects of insulin treatment in patients with AD [118–121]. However, it is important to consider that the mediating role of microglia in these studies remains to be established.

Further to manipulating microglial glucose metabolism, modulation through lipids has been also shown to affect microglial phagocytosis in vivo. Supplementation with oleoylethanolamide and its analogue KDS-5104 attenuated Aβ pathology in 5×FAD mice via peroxisome proliferator-activated receptor signalling [122]. Similarly, another bioactive lipid-mediator sphingosine 1-phosphate (S1P), which is associated with obesity, dyslipidemia, and insulin resistance, was found to regulate microglial phagocytosis [123]. S1P receptor 1 antagonist ponesimod was shown to reduce TLR4-induced neuroinflammation and enhance Aβ clearance in 5×FAD mice [124].

Finally, targeting the PI3K/AKT/mTOR pathway, which is a key regulator of energy balance and metabolism, appears to be an attractive strategy for altering microglial phagocytosis. Inhibition of SHIP1/2 (SH-2 containing inositol 5' polyphosphatase), which are upstream to PI3K/AKT, enhanced phagocytosis of Aβ and dying neurons by microglia isolated from pharmacologically treated mice [125]. Finally, microglia-specific deletion of Tsc1, a negative regulator of mTOR, resulted in mTOR activation, upregulation of TREM2, enhanced Aβ phagocytosis and improved cognition in 5×FAD mice [126].

Taken together, rapidly accumulating evidence supports the potential of microglial metabolic targeting to enhance microglial phagocytosis of Aβ (Table 3). However, it remains unclear if the beneficial effects of such interventions achieved via enhanced Aβ clearance are counteracted by unwarranted phagocytosis of healthy synapses. In the next section, we examine if and how metabolic manipulations affect the susceptibility of healthy neurons to microglial phagocytosis.

**Table 3:** Metabolic manipulations that enhance microglial Aβ phagocytosis in AD models

Manipulation	Mice model	Effect on phagocytosis	Proposed mechanism	References
Microglia-specific HK2 depletion	5×FAD	Promotion of Aβ phagocytosis in vivo and in vitro	Increase in microglial LPL; activation of lipid metabolism	[16]
Supplementation with flavonoid—sodium rutin	APP/PS1 and 5×FAD	Enhancement of Aβ phagocytosis in vivo and in vitro	Microglial metabolic switch from anaerobic glycolysis to mitochondrial OXPHOS	[109]
Anti-TLR2 tretment	APP/PS1	Reduced Aβ plaque burden in vivo, enhancement of Aβ phagocytosis in primary mouse microglia	Restoration of oxidative metabolism and reduced inflammasome activation	[110, 111]
Treatment with the NAD^+^ precursor -nicotinamide riboside	APP/PS1	Increase in Aβ phagocytosis in vivo	Reduced neuroinflammation, activation of cyclic GMP-AMP synthase (cGAS)	[113]
Insulin administration	3×Tg-AD and APP/PS1	Reduced Aβ load in vivo studies; enhanced Aβ phagocytosis under inflammatory conditions in vitro (BV2 microglia)	Reduction in inflammatory markers	[114–117]
Supplementation with oleoylethanolamide and its analogue—KDS-5104	5×FAD	Reduced Aβ pathology in vivo; depletion of PPARα and CD36 antibody pretreatment reduced Aβ phagocytosis in vitro	Upregulation of PPARα-CD36 axis	[122]
S1P receptor 1 antagonist -ponesimod tretment	5×FAD	Amelioration of Aβ pathology in vivo; enhancement of Aβ phagocytosis in primary mouse microglia	Increase in the IL-33/Stat6 signaling pathway	[124]
Selective loss of Tsc1, a negative regulator of mTOR in microglia	5×FAD	Amelioration of Aβ pathology; enhancement of Aβ phagocytosis in vitro	mTOR activation and upregulation of TREM2; increase in expression of CD68 and LAMP1 in Tsc1-deficient microglia	[126]

*PPARα* Peroxisome proliferator-activated receptor alpha; *GMP* Guanosine monophosphate

## The issue of pathological phagocytosis of healthy neurons: metabolic regulation of the neuron-microglia crosstalk

Microglial phagocytosis of healthy neurons as opposed to unhealthy or dying cells is a prominent feature in several NDDs. While phagocytosis of healthy synapses is crucial during experience-dependent sculpting of neuronal networks, excessive removal of live neurons and synapses is detrimental and contributes to neurodegeneration [12]. Such pathological phagocytosis can be triggered by the abnormal release of ‘find-me’ signals, over-expression of 'eat-me' signals or loss of ‘don’t-eat-me’ signals by healthy neurons [127].

‘Find-me’ chemoattractant signals can be released by stressed or injured neurons, as well as their neighbouring healthy neurons to induce microglial chemotaxis toward the site of injury. These include C-X3-C motif chemokine ligand 1 (CX3CL1), which chemoattracts microglia by binding to C-X3-C motif chemokine receptor 1 (CXC3R1) [128, 129]. Subsequently, altered CX3CL1–CX3CR1 signalling can have either beneficial or detrimental effects on cognitive functions depending on physiological vs. pathological contexts. A decrease in the CX3CL1–CX3CR1 signalling during physiological brain development may lead to the impairment of hippocampal cognitive function and synaptic plasticity [130–132]. On the contrary, such decrease could turn beneficial in neuroinflammation and neurodegeneration as it may decrease pathological synaptic pruning [133–135]. Notably, expression of ‘eat-me’ signals can be regulated by metabolic pathways. Obesity induced by high-fat diet in mice was associated with a decrease in the expression of CX3CL1 and CX3CR1 in the hippocampus and amygdala [130].

Another group of ‘find-me’ signals are nucleotides, such as ATP and uridine triphosphate (UTP). At high levels, ATP and UTP are readily sensed by microglial P2Y12 receptors and have been shown to promote microglial phagocytosis [127, 136]. Owing to the close association of intracellular and extracellular ATP and ADP levels with metabolic states, it is reasonable to assume that metabolic stimuli can have a strong impact on synaptic pruning via alteration of ‘find-me’ signals as well.

‘Eat-me’ signals, on the other hand, can be transiently expressed by stressed but viable neurons to instruct microglia for phagocytosis, or can be expressed by microglia themselves [127, 137]. One of the best characterized ‘eat-me’ signals is phosphatidylserine (PS), a phospholipid that under physiological conditions is localized exclusively to the cytoplasmic membrane leaflet [138]. However, under stressful conditions, PS translocates to the exoplasmic leaflet via calcium-activation of transmembrane proteins and translocases [139]. Additionally, the presence of oxidants or glutamate excitotoxicity also leads to enhanced expression of PS on the exoplasmic leaflet [140]. PS exposed on the neuronal cell surface is recognised by microglia via a number of receptors, such as adhesion G protein-coupled receptor GPR56 and TREM2 to initiate the process of neuronal phagocytosis [141, 142]. Additionally, PS can also bind opsonins such as growth arrest-specific 6 protein, ApoE, milk fat globule-EGF factor 8, and complement component 1q (C1q), which subsequently bind to microglial receptors [143–146]. Another ‘eat-me’ signal is calreticulin, which is similarly localised intracellularly but is expressed on the cell surface upon endoplasmic reticulum stress or inflammatory signalling [147]. Calreticulin exposure promotes neuronal phagocytosis by binding to microglial low-density lipoprotein receptor-related protein [148]. Finally, stressed neurons can also release a soluble ‘eat-me’ signal UDP, which binds to microglial purinergic P2Y6 receptor and promotes neuronal phagocytosis [149, 150].

Metabolic conditions can also affect the expression of ‘eat-me’ signals on the neurons. Notably, hyperglycemia is known to enhance neuronal intracellular calcium response to purinergic stimulation and thus may promote neuronal phagocytosis through increased expression of PS on the cell surface [151].

Finally, pathological phagocytosis of viable synapses and neurons can also be induced by the downregulation of ‘don’t eat me’ signals. One of the best-studied negative regulators of phagocytosis is the cluster of differentiation 47 (CD47) receptor [152–154]. CD47 is a transmembrane receptor that inhibits phagocytosis by binding and activating the transmembrane receptor signal regulatory protein α (SIRPα) on microglia [153]. Mice with a genetic deletion of CD47 display increased microglial engulfment of retinal ganglion cell inputs, excess pruning, and a sustained reduction in synaptic numbers [154].

Metabolic conditions, such as DM, have also been associated with CD47. Interestingly, contrary to its deleterious effects on microglial inflammatory responses and phagocytosis, DM was shown to increase the expression of CD47 in the hippocampus and prefrontal cortex in mice [155]. Similarly, another study showed that high glucose prevents the degradation of CD47, thus promoting its association with microglial SIRPα to suppress the phagocytosis of neurons [156, 157]. It is unclear if the increased expression of neuronal CD47 in DM is a compensatory step to fend off increased microglial phagocytosis or just a co-incidental finding.

In summary, similar to their effect on microglial phagocytic machinery, metabolic conditions can also impact the susceptibility of live neurons to microglia through their effect on neuronal ‘find-me’, ‘eat-me’, and ‘don’t-eat-me’ signals. This adds an additional layer of challenges when devising metabolism-focused strategies to preferentially enhance microglial clearance of Aβ in AD.

In the next section, we highlight the distinct and divergent aspects of microglial engulfment and digestion of Aβ as opposed to healthy neurons. A greater focus on the selective regulation of microglial phagocytosis of Aβ and healthy neurons may aid in elucidating strategies to preferentially enhance microglial clearance of Aβ.

## Towards specific regulation of microglial phagocytosis of Aβ vs. healthy neurons

As pointed out in the previous sections, concrete evidence supports the utility of at least three different metabolic manipulations in enhancing microglial phagocytosis of Aβ: activation of the PI3K/AKT/mTOR pathways, enhancing mitochondrial OXPHOS, and insulin.

While the effects of these interventions on microglial phagocytosis of Aβ vs. healthy synapses have not been studied in parallel, there is some evidence to suggest that the effects of PI3K/AKT/mTOR modulation on phagocytosis of Aβ vs. health neurons may be incongruent. Persistent activation of mTOR was associated with a reduction of microglial synaptic pruning in a translational study involving comparative analysis of autism spectrum disorder brains in mice and humans [158]. On the contrary, reduced mTOR-autophagy signalling has been associated with exaggerated microglial pruning of synapses [126]. Furthermore, activation of SHIP1, which is an upstream negative regulator of PI3K/AKT pathway, promotes phagocytosis of lipid-laden cargoes, such as synaptosomes and apoptotic neurons without affecting the phagocytosis of Aβ [159]. This raises the possibility of interventions, where PI3K/AKT/mTOR activators can be employed to preferentillay remove Aβ in aged brains where impaired synaptic pruning by microglia may be an acceptable side effect. Similarly, activating microglial OXPHOS in a stroke model via microglia-specific conditional knockout of Na/H exchanger enhanced synaptic stripping of injured neurons without substantially harming healthy neurons [126]. Hence, enhancing microglial OXPHOS could be another viable strategy to preferentially enhance microglial phagocytosis of Aβ via targeted therapies.

Furthermore, it is important to consider that the dynamics of engulfment and digestion of microglial phagocytic cargoes likely vary between Aβ vs. health synapses. During synaptic pruning, specific synaptic proteins and adhesion molecules on the surface of synapses play a pivotal role in marking them for elimination [160, 161]. Furthermore, neuronal activity patterns, in particular during development, determine the vulnerability of neurons to be engulfed by microglia for modeling neuronal circuits [162]. Conversely, the characteristic "find-me" and "eat-me" signals associated with Aβ are not exclusive. For example, complement proteins, particularly C1q, mark Aβ aggregates but also apoptotic bodies and synapses for recognition by microglia [163, 164]. Hence, microglial phagocytosis of Aβ could be combined with strategies that reduce the vulnerability of neurons against microglial phagocytosis, for instance, via upregulation of ‘don’t-eat-me’ signals or preventing elimination of synapses through modulation of their activity patterns. However, the stage of disease could play an important role in this regard. Aβ oligomers, which represent an early phase of pathology in AD, were recently shown to cause synaptic damage via neuronal overactivation. Microglial removal of such hyperactive synapses, shown to be mediated by PS-TREM2 signaling, could thus be beneficial in early stages of AD [165].

Finally, further evidence is warranted to delineate the dynamics of phagolysosomal digestion of Aβ vs. synaptic material in microglia. The inherent differences in the stability of Aβ deposits in comparison to synaptic proteins and myelin stipulate that the changes in the lysosomal environment, such as pH, may impact the digestion of these vastly different phagocytic cargoes. Therefore, understanding the disparate dynamics of microglial engulfment and digestion of Aβ vs. healthy neurons, as well as how they are affected by metabolism, holds tremendous promise for identifying ways to preferentially enhance microglial clearance of Aβ and other toxic deposits in disease contexts.

## Conclusions and future directions

In light of the reviewed evidence, it can be concluded that targeting microglial metabolism to alter their functions could be a viable preventive and therapeutic strategy in AD and potentially other NDDs. Microglial metabolism is closely related to their inflammatory responses, as well as phagocytosis. Therefore, modalities ranging from nutrient supplementation to targeting specific metabolic pathways via pharmacological or genetic manipulation can be envisioned to harness the power of microglia to clear Aβ deposits in AD. However, several challenges warrant critical consideration in this regard.

A foremost challenge is how to enhance microglial phagocytosis preferentially for misfolded proteins and dead cells with minimal harm to viable neurons. The reviewed evidence suggests that several metabolic manipulations could be exploited: notably, enhancing OXPHOS via supplementation with lactate, ketone bodies, PUFAs, flavonoids, or NAD^+^ can enhance microglial phagocytosis with relatively moderate or transient increase in their inflammatory responses. However, their effects on neuronal expression of ‘find-me’, ‘eat-me’, and ‘don’t-eat-me’ signals are not clearly known. There is some preliminary evidence that enhancing microglial OXPHOS as well as the PI3K/AKT/mTOR signalling may not severely impact healthy neurons. Nevertheless, this requires substantiation by robust comparative analyses of microglial phagocytosis of Aβ and healthy synapses after comparable metabolic manipulations.

Another challenge is related to the discrepancies in the observed effects of different metabolites on microglia. One of the potential underlying reasons for this could be the use of a variety of in vitro and ex vivo microglial models, which may be influenced by species-specific effects, the developmental stage, and the system used for generating immortalized cell line. Furthermore, it is important to consider that findings obtained in immortalized cell lines may not recapitulate processes occurring in vivo. BV2 microglia, immortalized using the v-raf/v-myc-carrying J2 retrovirus, for example, have been largely used for in vitro modeling of microglia. However, their proliferation rate and their morphology are very different from primary cells, and their transcriptomic and metabolic profiles are not always comparable [19, 166]. Therefore, the findings obtained using BV2 microglia should be interpreted with a cautious approach.

Additional confounding effects could be related to variable experimental designs. For instance, phagocytosis assays employed in existing studies reveal remarkable differences in the duration of experiments, type and concentration of nutrient manipulations, as well as substrates used to assess phagocytosis. This issue is further complicated by microglial phenotypic heterogeneity and lack of clear classification systems. Future investigations could be improved by conducting phagocytosis assays in microglia across multiple time points and use of different substrates in parallel (for example, checking the effects of a particular metabolic manipulation on phagocytosis of Aβ and synaptosomes simultaneously).

Finally, translating the findings from basic science studies to clinical settings remains a considerable challenge. Despite the emergence of evidence showing a clear implication of microglia in NDDs, assessments of microglia in studies involving post-mortem tissues from NDD patients remain infrequent. Furthermore, while amyloid scans are now routinely used to assess the progression of AD in clinical and research settings, modalities to assess microglial states and phagocytic activity are unavailable. Similarly, the findings from basic science studies performed in vitro or in rodent models are usually not substantiated by human validations.

Future investigations could benefit from a multi-pronged strategy where the key observations from experimental studies could be validated using a combination of patient-derived biological fluids, induced pluripotent stem cells (iPSCs), and imaging scans. For instance, to check the effects of a particular metabolic condition on microglial phagocytosis of Aβ, longitudinal studies that quantify amyloid and microglial states in parallel are possible. A combination of amyloid scans and TSPO, previously called peripheral benzodiazepine receptor imaging that indicates microglial inflammatory response could be helpful in this regard [167, 168]. Furthermore, stimulation of in vitro microglial models with patient blood or cerebrospinal fluid samples could provide further clues into how the functionality of microglia could be altered by specific metabolic conditions.

Finally, advances in the iPSC technology now enable the generation of microglia, as well as neurons from patient-derived material that could be used for comparative analyses after specific metabolic manipulations. Microglia generated from iPSCs reprogrammed from patient fibroblasts with *TREM2* mutations recapitulate the metabolic dysfunction associated with TREM2 deficiency in microglia. Notably, this inludes reduced mitochondrial respiratory capacity and impaired glycolytic switching [169, 170]. However, culturing of stem cell-derived microglia leads to an increased expression of glycolytic proteins causing a shift towards glycolysis [171]. A potential solution to address such glycolytic shifting in iPSC-derived microglia could be the xenotransplantation of microglia into the brain. Integration of human stem cell-derived microglia into the mouse brain has been shown to restore microglial homeostatic signatures on RNA-seq [172]. Similarly, transplantation of iPSC-derived microglia into 3D brain organoids can preserve their homestatic signatures and prevent the functional adaptations that can be induced by their culturing in vitro [173].

Based on a comprehensive evaluation of the relevant literature, it can be concluded that the metabolic flexibility of microglia as well as their dependence on specific metabolic pathways allows a unique opportunity in AD therapeutics. Optimizing metabolic approaches that preferentially enhance microglial phagocytosis of Aβ while limiting the susceptibility of healthy neurons to microglial phagocytosis and inflammatory outputs can allow us to harness microglia as an effective therapeutic tool in AD and potentially other NDDs.

## Abbreviations

AA: Arachidonic acid
ABCD transporters: ATP-binding cassette transporters subclass D
Acetyl-CoA: Acetyl coenzyme A
AD: Alzheimer’s disease
ADP: Adenosine diphosphate
ALA: Alpha linolenic acid
APOE: Apolipoprotein E
APP: Amyloid precursor protein
ATP: Adenosine triphosphate
Aβ: Amyloid beta
C1q: Complement component 1q
CD: Cluster of differentiation
CPT1: Carnitine acetyltransferase 1
CX3CL1: C-X3-C motif chemokine ligand 1
DHA: Docosahexaenoic acid
DM: Diabetes mellitus
EPA: Eicosapentaenoic acid
FABP: Fatty acid-binding protein
FAO: Fatty acid β-oxidation
FA: Fatty acid
GLUT: Glucose transporter
HK2: Hexokinase 2
IFNγ: Interferon gamma
IL: Interleukin
iPSC: Induced pluripotent stem cell
LDHB: Lactate dehydrogenase B
LPL: Lipoprotein lipase
LPS: Lipopolysaccharides
MCT: Monocarboxylate transporter
NDDs: Neurodegenerative disorders
NF-κB: Nuclear factor kappa B
OXPHOS: Oxidative phosphorylation
PD: Parkinson’s disease
PI3Ks: Phosphoinositide 3-kinases
PS: Phosphatidylserine
PUFA: Polyunsaturated fatty acid
ROS: Reactive oxygen species
SFA: Saturated fatty acid
SIRPα: Signal regulatory protein alpha
SR: Scavenger receptor
TCA cycle: Tricarboxylic acid cycle
TDG-PET: Fluorodeoxyglucose positron emission tomography
TLR: Toll-like receptor
TNF-α: Tumour necrosis factor alpha
TREM2: Triggering receptor expressed on myeloid cells 2
TSPO: Translocator protein
UTP: Uridine triphosphate
βHB: Beta-hydroxybutyrate
